# A Scoping Review on the Impact of the Environment on Racialized Immigrant Older Adults’ Social Connectedness and Sense of Belonging

**DOI:** 10.1155/jare/1089194

**Published:** 2026-02-20

**Authors:** Vivian Afrah Puplampu, Ashley Hai Yen Ho, Mashrur Kazi, Alberta Baffour-Awuah, Michelle Dalidowicz, Juliet Bushi, Jordana Salma, Joseph Osuji, Festus Y. Moasun, Mary Chipanshi, Florence Luhanga, Anahit Falihi, Christina Nsaliwa, Jordan Pierson, Leonie Mvumbi Mambu, Bukola Salami

**Affiliations:** ^1^ University of Regina, Regina, Canada, uregina.ca; ^2^ University of Alberta, Edmonton, Canada, ualberta.ca; ^3^ Saskatchewan Polytechnic, Saskatoon, Canada; ^4^ University of Windsor, Windsor, Canada, uwindsor.ca; ^5^ Mount Royal University, Calgary, Canada, mtroyal.ca; ^6^ Saskatoon Open Door Society, Saskatoon, Canada; ^7^ Edmonton Immigrant Services Association, Edmonton, Canada; ^8^ College Mathieu, Montfort Hospital, Ottawa, Ontario, Canada; ^9^ University of Calgary, Calgary, Canada, ucalgary.ca

**Keywords:** belongingness, Canada, environment, older adults, racialized immigrants, social connectedness

## Abstract

**Background and objective:**

The increasing number of immigrant and older adult populations in Canada is reflected among racialized groups. Migrating to a new country at an older age, language barriers, financial concerns and immigration policies present challenges for connecting to the community. Many racialized immigrant older adults (RIOAs) experience challenges related to engaging in and feeling a sense of belonging in the community. This scoping review maps out and summarizes evidence on the impact of the environment on RIOAs’ social connections and belongingness in Canada.

**Methods:**

Ageline, CINAHL, Medline (Ovid), APA PsycInfo, Sociological Abstracts, Joanna Briggs Institute EBP Database, the Cochrane Database and ProQuest Dissertation & Theses Global were searched for peer‐reviewed articles. Articles were included if they were published in English within the last 12 years, focused on RIOAs (non‐White and non‐Indigenous individuals aged 55 years and above) and reported on their connectedness and/or sense of belonging in Canada. Qualitative content analysis was used to code the data, the interpretation of which was guided by the social ecological model.

**Results:**

Thirty‐seven articles met the inclusion criteria, and five interrelated categories were identified: (1) Intrapersonal Level: RIOAs’ characteristics, expectations and choice of living arrangement; (2) Interpersonal Level: Meaning of social connection and experience of support; (3) Organizational Level: RIOAs’ experience with institutions in Canada; (4) Community Level: RIOAs’ experience in their neighbourhood; and (5) Public Policy Level: Poverty and retirement in Canada.

**Conclusion:**

Interconnected factors that affect RIOAs’ social connectedness and sense of belonging in Canada include personal characteristics, family status, the neighbourhood in which they reside and policy implications. Considering the aforementioned factors in programme and policy development may inform how to better support this population in Canada.

**Implications:**

A collaborative effort from family members, neighbours, community members, organizations and policymakers is needed to facilitate RIOAs’ engagement and sense of belonging in the community.

## 1. Introduction

Globally, the percentage of older adults is increasing. In 2023, older adults (65 years and above) accounted for 20% of the population in developed countries, 9% in developing countries and 3.7% in the least developed countries, with each expected to increase rapidly [1]. Older adults form 19% of Canada’s population [2]. Canada is a multicultural country, with 30% of its older adult population identifying as immigrants, of which 14% are racialized older adults [3–5]. Racialized immigrant older adults (RIOAs) can be defined as individuals born outside Canada who are non‐European, non‐White, non‐Caucasian and of non‐Indigenous descent and have been offered the right to stay in Canada permanently by immigration authorities through landed immigrant and refugee programmes [3, 6, 7]. RIOAs are a very diverse group who may self‐identify as Arab, Asian, Black from African countries, Black from the Caribbean, Black Canadian, Chinese, Central American, Central Asian, Japanese, Korean, Latin American, South Asian, Southeast Asian, Vietnamese and/or others, with the largest groups of newcomers arriving from Asia and Africa [3, 6]. The number of RIOAs in Canada is increasing, as those who migrated in their early twenties to middle age are ageing; more older adults are also arriving through family reunion [4]. With the increasing number of RIOAs in the country come challenges that many RIOAs encounter in engaging in the community, such as poverty and limited proficiency in the official languages [3, 8, 9].

Although their income level has increased, the general older adult population in Canada reports low income due to decreases in earnings at retirement [10]. Poverty in Canada is described using measures such as the Market Basket Measure (MBM), which is based on the cost of goods and services such as food, clothing, shelter and transportation [10]. When a person or a family’s disposable income is lower than the cost of these goods and services, the individual or family is deemed to be in poverty [10]. Poverty is exceptionally high among RIOAs, particularly women. Except for Japanese older adults, RIOAs are reported to have less varied sources of income compared to nonracialized older adults in Canada [4]. For example, Census 2021 data indicate only 38% of RIOAs had private retirement income compared to 66% of nonracialized older adults [4]. The low‐income level among RIOAs is associated with race/ethnicity, language, length of residency, limited private retirement income and limited working years that limit their eligibility for public pension benefits and social welfare benefits [4, 11, 12]. Additionally, according to Curtis et al. [11], most pension reciprocity agreements into which Canada has entered are with European countries, thus leaving many RIOAs from other countries ineligible for Canadian pension benefits. In Canada, older adults’ main sources of income are Old Age Security (OAS), Guaranteed Income Supplement (GIS), Canada/Québec Pension Plan (CPP/QPP), and employment‐based pension plans [11]. OAS and GIS are noncontributing public pensions available to Canadians or legal residents aged 65 years or above who have lived in Canada for 10 years or more after age 18 years [11]. CPP/QPP are funds to which working adults contribute and then access at retirement. The Government of Canada has increased its income support to older adults since 2016 through the OAS and GIS programmes to enhance their economic security [10]. For example, low‐income older adults who meet the criteria for pension benefits with an annual combined household income less than $41,616 receive the OAS maximum of $1409.72/month [13]. Additional funds of $667.41/month from the GIS are provided to couples of OAS recipients with low income ($29,712 annually). Some provinces such as Alberta provide other financial assistance to low‐income seniors and supplementary accommodation support if in care. In total, an older adult may receive between $2160 and $2950 per month. When the total pension received is compared to market expenditure for rent, food and transportation, this places older adults who depend solely on their government pension at risk of poverty. From 1996 to 2022, transfers from the government to older adults rarely increased, yet market income (income from employment, private retirement insurance and investment) increased by 65.9%, which is the main source of income growth for older adults in Canada. However, the precarious work in which many immigrants, including RIOAs, engage often does not offer them the opportunity to contribute to the CPP/QPP [14]. Furthermore, the minimum 10 years of residency requirement to access the OAS disqualifies many RIOAs from benefiting from the fund [15]. Although many RIOAs are retired, others still work to earn extra income to sustain themselves, and many RIOAs are unable to afford payments associated with attending social programmes that would enhance their social connections in the country [3, 8, 9, 12]. Most often, RIOAs have low income because of racial inequality experienced in the labour market and immigration policies [15, 16]. Older adults’ inability to work in lucrative and stable jobs threatens their financial security as well as their ability to engage in social activities that promote their social connections in the community [15]. Poverty has many consequences for RIOAs, including effects on their physical and mental health, as well as social connectedness and wellbeing. The terms connectedness and belongingness are sometimes used interchangeably. In this paper, we define social connectedness as the feeling of closeness facilitated by social connections, or the proportion of an individual’s social network with which they are in regular contact, which for older adults typically comprises their spouse, children, siblings and friends [17]. The convoy model highlights the links between the quantity and quality of social relations and the protective factor offered to the older adult to promote health, including reduced mortality [17, 18]. In the convoy model, the individual is positioned at the centre of their social relations, with whom the person shares their perspective, and the definition of social relations recognizes the multiple forms of social relations or contacts an individual might possess. Individuals closer to the circle are people older adults consider important, i.e., that they cannot live without, while those that occupy the outer part of the circle they can live without [17]. Children remain important to older adults and are closer to the circle [17]. The convoy model further identifies individual characteristics, including age, gender, socioeconomic status and race/ethnicity, as well as situational characteristics, such as the contexts in which people live, including cultural context and historical events, that play a role in the ability to promote or constrain a person’s ability to create a social network. Meaningful social connections or social relationships with contacts such as family members and friends are the main medium for experiencing a sense of belonging, i.e., being accepted and valued, while deprivation of social and physical contact may reduce the sense of belonging [19].

We define *social belonging* as an individual’s perception of feelings of closeness, having and maintaining positive relationships with others, being secure and being part of a community [20–24]. Humans are social beings who instinctually want to form and maintain relationships, be accepted, be loved and feel included in a community [14, 20]. Belongingness is a fundamental human need that all people want to satisfy [25]. Allen and colleagues defined belonging ‘as a subjective feeling that one is an integral part of their surrounding systems, including family, friends, school, work environments, community, cultural groups, and physical places’ [25] p. 88. Belonging exists because of and in connection with the systems in which people live [25]. The precursor for a sense of belonging is relationships with family members and friends through frequent interactions and concerns for each other’s welfare [19]. Engaging in meaningful activities, including participation in family events, supporting each other via grandparenting and engaging with friends in cultural or sports activities or going out for meals, is an exchange of affection that promoted older adults’ sense of belonging prior to the COVID‐19 pandemic [19]. Derrer‐Merk et al. [19] discussed how meaningful social connections must exist for belonging to be enacted. Sense of belonging is enacted by frequently meeting family members and friends, spending time together in person and enjoying each other’s company through activities such as having social contacts in proximity, exchanging support and expressing concern for each other’s welfare [19, 26]. Enacted belonging provides a protective effect in times of distress, such as during the COVID‐19 pandemic [19]. During the COVID‐19 pandemic, physical contact was restricted and many older adults had their sense of belonging challenged as they were unable to physically meet their social networks face to face [19].

Baumeister and Leary​ [20] and Derrer‐Merk et al. [19] posited that belongingness is a need that when not fulfilled can lead to isolation, feelings of depression, loneliness and challenges in resilience. The need to belong is a fundamental human motivation that people strive to meet by maintaining a minimum number and quality of social networks. Allen et al. [25] explained that belonging is an individual’s subjective feeling of connection to a chosen group, place and/or culture and a dynamic interaction with the social environment. Allen et al. [25] proposed an integrative framework on belongingness that posits four interrelated factors are needed for a person to feel they belong in their environment: the individual’s competency, such as knowledge and skills for belonging; opportunity given to belong to a group; motivation to belong by being accepted by the group; and perceptions of feeling they belong in the group [25]. Allen et al. [25] further discussed how trait belonging, which is the lasting sense of belonging, is essential for mental health and wellbeing. In contrast, a state or situational form of belonging is a temporary feeling of belonging based on one’s thoughts, feelings and beliefs. For example, an older adult could measure their sense of belonging to a group, such as a church group, based on support received from the church [19]. Other benefits of belonging to older adults include strengthened resilience and improved mental wellbeing [19]. Additionally, among students, feeling they belong contributes to better academic performance and improved health, positive social relationships, occupational success and improved physical and mental health [25]. A study by Savas et al. [26] with international retirement migrants from the Netherlands found that the older adults’ sense of belonging in the destination country protected them from social loneliness. Lack of social connection will lead to social loneliness, which is a health risk. Migrant older adults feel lonely because of challenges associated with making new friends, and their ability to maintain contact with relations in their home country and establish new relations in the new country will determine their experience of loneliness (subjective evaluation of inadequate social relations). Moreover, retirement migrants have typically been connected to their country of origin for most of their lives and might want to keep their own cultural identity after migration [26]. Feeling a sense of belonging through frequent contact with neighbours in the destination country can reduce the risk of social loneliness [26]. Despite the identified benefits of belonging to humanity, challenges to feeling a sense of belonging are particularly seen in minorities and other groups who are historically marginalized by mainstream cultures [25]. As such, belongingness might be a need that many RIOAs in Canada struggle to fulfil. Being a member of one group can satisfy the need to belong and reduce the need to belong to another group [20]. The ability of RIOAs in Canada to engage in at least one group can meet their need to belong if the group fulfils the indicators for belonging, i.e., experiencing reciprocal care (being cared about while caring about others), caring for others, frequent personal contact, belonging to a group or community and being accepted in a community [20–24]. Social belonging plays a crucial and beneficial role in the lives and wellbeing of RIOAs, as it combats loneliness, enhances access to resources, provides a sense of safety, betters health and improves quality of life [27, 28]. Kansanga et al.’s [22] research in Canada highlights the strong influence of sense of belonging in ensuring food security among older adults due to the network of social relations available to individuals, who often will provide support to others including sharing of food when needed. Furthermore, a sense of belonging reduces inflammation markers and alleviates the onset of dementia and depression in older adults [23]. While the benefits of social connectedness and belonging are established in the literature, the synergistic effect of factors including language barriers, policies in the receiving country, RIOAs’ connection to the country of origin, cultural identity and risk of poverty in Canada disadvantages them from engaging in social programmes.

Using the social ecological model (SEM), gerontological researchers have examined the interactions that occur between older adults and their environment and evaluated how interactions with the environment influence older adults’ ability to function, age in place and connect with the community [29–31]. We draw from the human ecological model to enhance understanding of the multilayered and interrelated levels of the environment on people’s interaction with the environment they live [32–34]. Bronfenbrenner [32] designed the human ecological model to study child development and proposed five socially organized systems that support human growth starting from the microsystem (relationship between a person and the immediate environment such as family), mesosystem (linkages between two or more microsystems), ecosystem (relationships with systems that do not involve the person but have impact on the individual), macrosystem (institutional pattern of culture, belief systems on the person) to chronosystems (change over the life course of the person and in the environment). The human ecological model recognizes that people do not live in isolation but are influenced by their physical, social and environmental context [34]. The SEM gives greater attention to the social, institutional and cultural contexts of the interaction between people and their environment than the human ecological model, which focuses on biological processes and the environment; instead, the SEM examines the interplay between the different levels of the environment and their influence on people’s wellbeing [33]. Social–ecological theorists’ conceptualization of the environment considers the social, physical, cultural and political components that shape people’s behaviour and lives [35] and will be the conceptual definition used in this review.

The *social environment* includes forming relationships with others and those with wider social institutions, and includes five categories: intrapersonal, interpersonal, organizational, community and public policy [20, 29–31, 33, 36]. The intrapersonal level involves older adults’ personal characteristics, such as health status, motivation, language proficiency, gender, age and knowledge, which impact their interactions with people who provide support and companionship [20, 29–31, 36]. The interpersonal level includes older adults’ interactions with people such as family members and friends that form their social network, promote social integration and engagement, and provide social support [29, 31]. The organizational level involves settings or institutions with operational rules and regulations with which older adults may or may not have direct contact but that still impact their wellbeing, such as the older adult’s or their adult child’s work environment. The community level involves relationships among organizations and the sociostructural qualities of the settings, such as neighbourhoods, which are crucial for social cohesion, physical wellbeing and emotional wellbeing among members [31, 33]. The public policy level describes the larger societal and sociocultural attributes of the host country, such as immigration policies, laws, customs and language that influence older adults’ social engagement in the society. In this review, we added to the public policy level the resources and social support provided by the host country, including transportation and financial support, as well as health and social services that contribute to older adults’ engagement in the community [19, 20, 29–31, 36].

The *physical environment* constitutes weather, spaces, sidewalks, bus routes, parking and infrastructure with which people directly or indirectly interact [12, 30, 36, 37]. Environmental factors such as absence of culturally relevant activities in the neighbourhood, unsafe neighbourhood and absence of accessibility in the built environment negatively impact older adults’ social connections due to the influence of these factors on interactions with people (family members, friends) and engagement with institutions [38]. A study on the impact of the environment on racialized older adults in the United States indicated that Black older adults with high annual income ($50,000 and $75,000), advanced education and employment were more engaged in social groups [38]. Strong engagement in the neighbourhood and neighbourhood cohesion is associated with participants’ social connectedness and sense of belonging, while neighbourhood physical disorder such as vandalism, drugs and crime, and fear of walking in the dark is associated with social isolation among older adults [38]. Similarly, Grenier et al.’s [12] study in Canada indicated that, due to their characteristics, older adults might be fearful of leaving their homes when confronted by neighbourhoods with drug use, crime or snowy sidewalks. Gerontological literature in Canada provides evidence that RIOAs experience differential treatment during their life course and retirement that precipitates the group’s exclusion from social programmes in society; many RIOAs, particularly recently arrived immigrants (< 5 years in the country), live in sketchy neighbourhoods, while established RIOAs struggle with poverty [12].

While some research has been conducted on the impact of the environment on older adults’ social connections in Canada, there remains a notable paucity of research on the impact of the social, political and economic environments of the country on RIOAs’ social connectedness. While some studies have explored social dimensions of wellbeing such as loneliness in older immigrants in Canada [39, 40], our scoping review aims to use the SEM to focus on social connectedness and sense of belonging, specifically in Canadian RIOAs. Thus, the purpose of this review is to identify the scope of the current literature on the impact of the environment on RIOAs’ social connectedness and sense of belonging in Canada and to reveal gaps in knowledge on the topic. This review will shed light on the support needs of RIOAs and areas for future research with this population.

## 2. Methods

This review was conducted in accordance with the Joanna Briggs Institute (JBI) methodology for scoping reviews and the Preferred Reporting Items for Systematic Reviews and Meta‐Analyses extension for Scoping Reviews (PRISMA‐ScR) [41]. A scoping review is a type of knowledge synthesis that systematically identifies and maps the breadth of evidence available on a topic, field, concept or issue [42, 43]. Scoping reviews are descriptive and often address broader questions than systematic reviews [42, 43]. Questions such as what is known about a particular concept are best answered using a scoping review method [42, 43]. The research team chose a scoping review instead of a systematic review because of the broad nature of the question guiding the review as well as the absence of any review on the impact of the environment on RIOAs’ social connectedness and belonging in Canada. Hence, we selected a scoping review to map out the research done in this area and identify any gaps in the literature to inform future research [42, 43]. The JBI scoping review methodology includes having a research question and aligned objective, developing the inclusion criteria, searching the literature, selecting the evidence, extracting data, analysing the evidence, presenting the results, summarizing the evidence and consulting experts, which can be optional [41]. This scoping review was further guided by the SEM framework, which permitted the identification of the impact of multiple environmental layers on RIOAs’ social connections in Canada [29, 31, 44].

### 2.1. Review Question

The question that guided the scoping review was as follows: What is the extent of the literature and evidence on the impact of the environment on RIOAs’ social connectedness and sense of belonging in Canada?

### 2.2. Inclusion/Eligibility Criteria

(a) Population: For this scoping review, *older adults* were defined as individuals aged 55 years and above. We recognize age 65 is the widely accepted cut‐off for older adults in Canada, e.g., as used in the introduction of this paper; however, 55 years was selected as the cut‐off age in this review because it aligns with the definition of older adults in the literature on racialized populations [45, 46]. Participants must also self‐identify as racialized immigrants and have lived in Canada for more than 6 months. We use the term *immigrants* to refer to individuals born outside Canada to non‐Canadian parents and include the following groups: landed immigrants (also called permanent residents, who came through the economic or family reunification class), refugees (individuals seeking Canada’s humanitarian protection), diaspora, newcomers and foreign workers [45, 47]. Some studies were excluded even though they focused on related topics such as social inclusion [48], as they were not set in Canada, where the literature on racialized older adults is sparse and social and migration policies for older adults can significantly differ from other immigrant‐receiving nations [5]. (b) Concept: social connectedness and sense of belonging were the target concepts. Articles describing how the environment impacts the social connectedness and sense of belonging of RIOAs in Canada were included. Articles were excluded if they focused on RIOAs’ health conditions, diagnoses and access to services but did not discuss the impact of the environment on social connectedness and sense of belonging or did not name a racialized group involved in the study. (c) Context: The context of the review is Canada. The data extraction table includes information on the geographic location of RIOAs in Canada. (d) Types of Sources: We searched databases comprising mainly primary studies, including quantitative and qualitative studies, published in peer‐reviewed journals that met the inclusion criteria. Databases containing grey literature, such as Google Scholar, Google, Grey Matters Database (CADTH), SSRN and DOAJ, were systematically searched. However, no new articles that met the review inclusion criteria were identified. Sources such as websites, blogs, magazines and opinion papers were excluded.

### 2.3. Search Strategy (Searching the Literature)

A three‐step search strategy was utilized to identify both published and unpublished studies. First, two health science librarians conducted an initial limited search of MEDLINE (Ovid), CINAHL (EBSCO), Embase, JBI EBP Database, Cochrane and Google Scholar to identify articles on the topic. No review articles were found in the JBI search. Words in the titles and abstracts of relevant articles and the index terms that described the articles were used to develop a complete search strategy for APA PsycInfo, Sociological Abstracts and ProQuest Dissertation & Theses Global (see Appendix 1). The review was registered in the Open Science Framework (OSF) on 26 August 2024. The search strategy, including all identified MeSH headings, keywords and index terms, was adapted for each database and/or information source.

Only studies published in English were included because most of the research team members are fluent in English as a primary language. Studies published from January 2013 to 2023 were included to align with the increasing population of ageing racialized people in Canada in the last decade. A second search was completed in 2024 and a third search was conducted in the Ageline database in July 2025 to obtain recent literature on the topic, with only two additional articles found that met the inclusion criteria. The 12‐year timeframe of our search strengthens the relevance of our scoping review findings for policy and future research.

### 2.4. Source of Evidence (Selecting Studies)

Following the search, 969 identified citations were collated and uploaded into Zotero, which was used to remove 185 duplicates. Three independent reviewers screened 784 titles and abstracts in JBI SUMARI [41, 49] for assessment against the inclusion criteria, with 710 articles excluded. While the 2018 JBI framework recommends pilot testing the title, abstract and full‐text screening for inter‐rater reliability at each stage of the study selection [43], the 2024 knowledge synthesis guidelines do not include this requirement [50]. Our review team omitted this activity, but to increase consistency among team members, we screened about 15 titles and abstracts independently and met to discuss the results before continuing with the remaining screening independently [43]. A total of 74 relevant sources were retrieved. The first author screened 50% of titles, abstracts and full‐text articles, the third author screened 70% of titles and abstracts, and the second author independently screened the full text of the selected citations against the inclusion criteria. Reasons for exclusion were recorded. Any disagreements among the reviewers at each stage of the selection process were resolved through discussions. A total of 37 articles were ultimately included in the review. The results of the search and the study inclusion process are presented in a PRISMA flow diagram (see Appendix 2). Although not generally required for scoping reviews [43, 49], we conducted a critical appraisal of individual sources of evidence conducted in JBI SUMARI and found the studies were of medium to strong quality.

### 2.5. Data Extraction

Five independent reviewers extracted data from the included studies using a data extraction tool from JBI SUMARI and adapted by the reviewers [43]. The extracted data included specific details about the RIOAs, environment, RIOAs’ social connectedness and sense of belonging in Canada, study methods and key findings relevant to the review question (see a draft extraction form in Appendix 3).

### 2.6. Data Analysis and Presentation of Results

Qualitative content analysis was employed to categorize information from the extracted data [42]. Pollock et al. [42] discussed how data analysis in scoping reviews is descriptive and that qualitative content analysis is sufficient to identify the characteristics and factors related to a concept being mapped out. Basic qualitative content analysis involves categorization to map out the characteristics of the results to address the scoping review question [42]. Categorization involves three phases: preparing, organizing and reporting the data [42]. During the preparation stage, an inductive or deductive approach can be employed. An inductive approach is used when there is limited evidence on the topic or the goal of the review is to inform a conceptual framework or theory, while a deductive approach is used to map out data to an established framework or theory in the literature [42]. Pollock et al. [42] discussed how both approaches could be used in a scoping review, which is the case for this review. Data analysis began inductively, and then, categorization was guided by the SEM framework. In the organizing phase, the review team familiarized themselves with the data through reading and open coding to assign labels to describe the data. Data were categorized to describe what is happening, and a coding framework was formed through an inductive process. Two review team members separately went through the extracted data and assigned a category, then came together to assess any discrepancies in the categorization. We then employed the SEM to further group the data into intrapersonal, interpersonal, organizational, community and public policy levels. During this deductive phase, the two members of the review team went through the data to ensure they reflected an understanding of the SEM framework [42].

The reporting phase of the qualitative content analysis involved using the PRISMA‐ScR checklist (Appendix 4) to indicate sections in the manuscript where components of the review are located [42]. We also used a data extraction table to present the data; no other visual presentation approach, such as a word cloud, was employed [42]. A narrative summary accompanied the charted results to describe how the results relate to the review objective and question [42].

## 3. Results

The initial search identified 969 articles. After removing duplicates, 784 titles and abstracts were screened and 710 articles were excluded. Seventy‐four relevant full‐text articles were retrieved and assessed for eligibility, of which 37 articles published between 2013 and 2025 were included in the review: 12 were quantitative studies, 24 were qualitative studies, and one was a synthesis study. All studies included in the review were conducted in Canadian cities with large immigrant populations, such as Toronto, Vancouver, Montréal, Edmonton, Calgary and Saskatoon. The impact of the environment on RIOAs’ social connection and belonging was organized based on levels in the SEM. The identified categories were as follows: (1) Intrapersonal Level: RIOAs’ characteristics, expectations and choice of living arrangement; (2) Interpersonal Level: Meaning of social connection and experiences of support; (3) Organizational Level: RIOAs’ experience with institutions in Canada; (4) Community Level: RIOAs’ experience in their neighbourhood; and (5) Public Policy Level: Poverty and retirement in Canada.

### 3.1. Category 1 Intrapersonal Level: RIOAs’ Characteristics, Expectations and Choice of Living Arrangement

This category is about RIOAs’ values, health context and knowledge that influence their choice of living arrangement and shape their social interactions with the community. Many of the RIOAs in the studies included in this review were born outside Canada, were 55 years and above and may have a health challenge. A common value revolved around the concept of filial piety, which relates to the expectation of RIOAs that their adult children will take care of them [21, 51]. Filial piety influenced some RIOAs’ choice of living arrangement [27, 51–54] and acceptance of support from outsiders [51, 55–58]. Many older adults who adhered to filial piety norms (e.g., Chinese, South Asian and Black) were uncomfortable receiving support from an outsider [55, 56, 58]. Although some older adults reduced their expectation that their adult children would care for them so as not to be a burden to them, filial piety was common among racialized groups, such as Arabs, Chinese, people of African descent, East Asians and South Asians, and influenced their choice of living arrangement [51, 55, 59, 60]. The great emphasis on filial piety reflects the importance of cultural values to the older adults and how it shapes their social connectedness and feeling of belonging in their destination country [26]. Older adults’ sense of belonging includes attachments to people, social groups, places, identities and values. Savas et al. [26] discussed how retirement migrants are connected to their country of origin, where they spent most of their lives and might want to maintain their own cultural identity after migration. Under this category are subcategories of RIOAs’ living arrangements and language barriers.

#### 3.1.1. RIOAs’ Living Arrangements

Many RIOAs, particularly those from South Asia (India, Bangladesh, Pakistan, Sri Lanka, Nepal, Philippines) and other Asian countries (China, Japan), lived in multigenerational households with their adult children and their families [53, 57]. For example, in Luo’s [52] study on Chinese older adults in Canada, the majority (66.6%) lived with their spouses, children and grandchildren, while 32% lived independently. Some older adults found themselves negotiating accommodations with their children because of the lack of affordable and accessible housing [53, 59–61]. In a multigenerational living arrangement, some older adults, particularly women, practised reciprocity by engaging in household chores and caregiving duties for their grandchildren, which might affect their ability to socialize outside the family because of the limited time available for social programmes outside the home or the absence of support provided to the older adult such as transportation to attend the events [21, 54, 61–64]. Some older adults received a meagre supplemental income by providing childcare in the community to assist their adult children with the cost of living [61]. A weakness of multigenerational living arrangements is the reported incidence of emotional and financial abuse of older adults and the accompanying loss of the older adult’s autonomy [54, 65]. Key challenges experienced in mainstream continuing care settings for older adults include language barriers, inequitable care provision, racism, voicelessness and ageism, all of which impact their social connectedness and sense of belonging [27, 52, 66].

Some older adults lived independently, either with or without their spouses, while a small number lived in assisted living facilities for older adults [52, 57]. Many RIOAs who had been living in Canada for a longer time lived in independent housing arrangements separate from their adult children [65, 67]. Ng and Northcott’s [68] study with South Asian older immigrants in Edmonton reported that those who lived alone felt lonelier than those who lived with family members. A small number of studies commented on the physical environment, such as challenges with housing, including those associated with accessing or maintaining it, as well as challenges associated with weather, such as difficulty walking in the snow that limited their ability to go out to attend programmes [52, 61, 69]. Some older adults commented that challenges with mobility or health prevented them from going out or participating in programmes [15, 69].

#### 3.1.2. Language Barriers

Linguistic ability can also determine whether older adults will engage with society in their destination country. Language barriers not only make it hard for racialized older adults to feel connected to their community but also reduce their access to essential services such as housing and healthcare [69]. Language barriers also lead to increased experiences of discrimination and impact quality of life in the destination country [28, 70–72]. Dhillon and Humble [55] found 56% of RIOAs only understood a few words in English—one of the two official languages in Canada—thereby limiting their access to services and social connectedness. While some RIOAs were well educated and fluent in at least one of Canada’s official languages, many studies included in this review recruited their participants from settlement and ethnocultural organizations whose older adult clients experienced language barriers that led to racist experiences [15, 69].

### 3.2. Category 2 Interpersonal Level: Meaning of Social Connectedness and Experience of Support

Participants from the studies reviewed define social connectedness in the context of sense of belonging as the older adults’ experience of happiness and sound mental health [73, 74], social networks and close ties [21, 63, 65, 75], receiving and giving support [51, 76], integration and sense of belonging [27, 55, 59, 63, 77, 78] and social connectedness to the community [79]. Words that described lack of voice [84], limited social connections and loneliness were also used [67, 80]. While cultural differences might exist among various ethnic groups, such as Black Caribbeans, Black Canadians, Blacks from African countries, Chinese, Japanese, Korean, Middle Eastern and South Asian, and might influence their experiences of social connectedness and sense of belonging, shared similarities included experiences of marginalization, dependence and reduced financial and social capital that impact their social connectedness. Longer length of stay in Canada positively correlated with some RIOAs’ sense of belonging, which suggests the increased likelihood of negative experiences for more recently arrived RIOAs [15]. A subcategory under this category is RIOAs’ support network.

#### 3.2.1. RIOAs’ Support Network

RIOAs reported their greatest sources of support and social contact were family members, ethnocultural communities, religious communities and volunteering opportunities that enhanced their sense of belonging in the country [53, 68]. The group’s most significant source of sense of belonging came from family members, particularly adult children and grandchildren, who visit, call, take them out and provide care in some situations [47, 68]. RIOAs with positive family relationships felt less lonely and were happier within their close circle of family members and friends [53, 68]. However, older adults’ social lives changed as family members and friends passed away [65]. Similarly, social services, settlement services and religious institutions such as churches and mosques were essential in enabling RIOAs to establish new friends and social support systems [75]. However, older adults faced challenges attending social programmes for a number of reasons, including transportation, lack of time due to their family caregiving roles and absence of a support person to accompany them to the programme for those with mobility issues [12, 15, 75]. Ferrer et al. [21] reported that the Filipino community provided intergenerational care to older Filipino adults in Canada with whom they had no blood relationship (fictive kin) to help the older adults have a sense of belonging in Canada, and that the older adults reciprocally provided caregiving.

### 3.3. Category 3 Organizational Level: RIOAs’ Experience With Institutions in Canada

This category describes institutions with rules and regulations, such as the labour market and healthcare system, and reflects on how they shape RIOAs’ social connectedness and sense of belonging in Canada. Subcategories under this theme are structural racism and mental health.

#### 3.3.1. Structural Racism

The included literature revealed some RIOAs experienced challenges such as individual and systemic racism in the community due to cultural and linguistic differences [15, 27, 28, 55, 81]. Changes in individuals’ identities might be a consequence of racism. For example, McAlpine et al. [28] and Ferrer et al. [64] reported that older refugees in Canada experienced more racism than settled RIOAs due to language barriers, particularly when accessing services such as healthcare. The RIOAs depended on family members and settlement organizations to schedule appointments, sometimes restricting their use of services and participation in social engagements in the community [27, 28, 55]. Furthermore, because the population experienced financial challenges, some RIOAs continued to engage in employment and reported experiencing racism at work. Many RIOA workers, particularly live‐in caregivers or domestic workers, reported being physically or emotionally abused [64]. Additionally, many RIOAs were unable to work in jobs that aligned with their academic qualifications or professional experience because they possessed foreign degrees and had limited Canadian experience; many endured significant deskilling to ultimately find work in Canada, contributing to their experience of poverty and ageing alone in Canada [64, 77]. Degrees and credentials from certain countries were not recognized, contributing to RIOAs’ inability to find jobs or forcing them into precarious jobs that had no pension plans or health benefits, thus contributing to financial vulnerability [64, 76].

#### 3.3.2. Mental Health Concerns

The interplay of factors including immigration laws, racism and poverty contributed to anxiety and feelings of insecurity among some racialized older adults, such as Black older adults, and increased their need for mental health support [65]. Culturally, many people from racialized populations do not report issues with mental health because of negative labels assigned to mental illness [65]. RIOAs who experienced racism had a 50% chance of reporting poor health, social isolation and loneliness [28]. Mental health was a concern among RIOAs, though many do not report symptoms or seek help [57, 74, 82]. The reduced reporting of mental health issues such as depression among RIOAs leads to the under‐treatment of mental illness in this group, negatively impacting family dynamics and the older adults’ interaction with the community [74]. Some contributing factors to mental health issues among RIOAs include trauma, racism, migration, immigration law promoting dependence, reduced social contact with friends, small social networks and support systems, language barriers, poverty and conflicts, including those with family and others, which intensify grief and their social disconnection with the community [51, 55, 79, 81]. Peer‐based interventions, where an older adult is paired with another older adult, were effective in reducing social isolation and loneliness among racialized older adults, improving their mental health overall [73].

### 3.4. Category 4 Community Level: RIOAs Experience in the Neighbourhood

This category describes the RIOAs’ experiences with social cohesion, social connections and sense of belonging in their immediate community, such as their neighbourhood in the receiving country. Subcategories are limited sense of community ties, social isolation and loneliness.

#### 3.4.1. Limited Sense of Community Ties

Many racialized older adults had no friends outside their family circle and reminisced about their home country [55]. Many RIOAs expressed having limited social contact due to a lack of extended family in Canada, linguistic and cultural differences, and racism [55, 70, 77]. Many also shared they do not experience the village lifestyle in Canada, where everyone knows each other; instead, they are strangers to people in their neighbourhood and community, and people are strangers to them, and, as a result, many RIOAs experience loneliness [55, 81]. The cultural differences that RIOAs experience in Canada can impact their social connectedness, community involvement and sense of belonging [59, 60]. Differences in cultural values, lack or reduced number of familiar people and familiarity in the context older adults had lived for most of their lives prior to migration contribute to their feelings of homesickness in the new country [26]. Adapting to new cultural values, struggling to make new close friends and losing contact with close ties such as children and close friends from their home country can be challenging for migrant older adults and lead to loneliness. For those who lacked prior connection to Canada, the lack of time to establish connections in the community resulted in social isolation [59, 60].

#### 3.4.2. Social Isolation and Loneliness

Many RIOAs established connections solely with friends from the same culture to help enhance their sense of belonging in the host country [27, 71]. Excessive dependence on family contributed to social isolation because the older adults were less likely to attend social functions and may experience suffering and conflict from only being in contact with their family members. Besides family, RIOAs benefitted from gatherings where they spoke their language and ate cultural food [83]. This concept is called the ‘social enclave’, the main purpose of which is to give the older adults more linguistic proximity to each other and bring the community together as well as develop resilience, particularly against racism [27, 71]. The social enclave provides a benefit to older adults by connecting them to their cultural group, maintaining identity and enhancing feelings that they belong. Disadvantages of the social enclave are limited contact and interaction with the larger society [27, 71]. Implications of the social enclave include emotional support for the older adult and a source of social capital, but also the potential to lead to isolation from the mainstream community. While other studies among migrant immigrants have reported strong emotional belonging when older adults have meaningful relationship with family members and close friends [26], research among racialized older adults in Canada indicates older adults who often depend mainly on family members in the host country for their social needs report feelings of loneliness because their adult children spend limited time in engaging with them as they often work excessively [27, 71]. Hence, volunteering helps older adults find a role in society and reduces feelings of loneliness [53, 67]. However, many RIOAs did not have the finances or support to attend programmes in the community due to different experiences with institutions.

### 3.5. Category 5 Public Policy Level: Poverty and Retirement in Canada

Poverty is prevalent among RIOAs in Canada due to the intersection of immigration law, race, age of migration, language, gender and the absence of Canadian education and work experience, which often results in deskilling [15, 61]. Newcomer RIOAs and those who have not engaged in the workforce postmigration were reported to be barely able to provide food and clothing for themselves and live in deplorable housing conditions [61]. Some newcomer RIOAs shared living space with adult children as they were unable to rent a place on their own [53, 61]. This means that many RIOAs, particularly newly arrived older adults, are vulnerable and depend more on sponsors such as adult children and society to connect them to the Canadian community and feel a sense of belonging. The subcategories under this category are limited income and retirement and pensions.

#### 3.5.1. Limited Income

One of the biggest reasons older adults struggled to maintain financial security is the limited income they receive. Many found themselves in jobs that lacked pensions and other employee plans [15, 64] because they entered the workforce in Canada later in life [61, 64]. One study revealed that seven out of 10 Filipinos in the Greater Toronto Area lived in a state of economic insecurity or poverty [61]. Similarly, Hepburn’s [15] study with Jamaican Canadians in the Greater Toronto Area found that the older adults were vulnerable and at risk of poverty. Poverty and financial struggles had a detrimental impact on older adults’ social connectedness and sense of belonging by taking away their sense of security. Many found housing to be a significant issue because their pension income was insufficient to repair damage such as leaks, faucets and stoves [76].

#### 3.5.2. Retirement and Pensions

While many RIOAs looked forward to retirement in Canada after working all their lives [64], many were unaware of pension benefits and eligibility criteria and thus live in poverty at retirement [15]. Many OAS financial programmes (e.g., OAS and GIS) require applicants to be 65 years or older and to have lived in the country for at least 10 years, making many RIOAs ineligible [55, 61, 76]. Immigrants also need to have worked in Canada to benefit from the CPP or QPP. The various ageing‐supportive resources contributed to their excitement about retirement in Canada; however, not being eligible to access these resources predisposed them to poverty, marginalization and limited involvement in the community [12, 64, 84]. Racialized older adults appreciated the different types of support in Canada including healthcare [84] and financial support from the government and wish to benefit from them, but this can be particularly frustrating for newly arrived RIOAs who are unable to access these supports.

## 4. Discussion

This scoping review aimed to identify the extent of the literature on the impact of the environment on RIOAs’ social connectedness and sense of belonging in Canada. The key finding is the heightened interplay of multiple factors that shape the experiences of RIOAs and negatively influence their engagement in Canadian society. This review used the SEM to summarize the available studies on the impact of various facets of the environment on RIOAs’ social connectedness and sense of belonging in Canada. Multiple dimensions of the physical environment, such as geography, culture and policy, interplay with the older adults to influence their wellbeing. The social environment is grouped under five levels: intrapersonal, interpersonal, organizational, community and policy levels in the SEM. The intrapersonal level examines how personal characteristics such as values, socioeconomic and health status, and language proficiency influence RIOAs’ engagement with the general Canadian population. While their perception of the role of their children in taking care of them influenced the selection of living environment, RIOAs’ socioeconomic status shaped their choice of accommodation. Many newly arrived older adults live with and depend on their adult children and grandchildren for support, including transportation to facilitate social connections. Family members are a key support system for RIOAs, but their schedules limit their ability to adequately support RIOAs’ social inclusion [12, 69]. Furthermore, RIOAs engage in housework and retirement employment to support themselves, limiting time for social engagement. Although supporting adult children to raise grandchildren is a form of meaningful social connection for older adults and enforces belongingness [19], in the context of the RIOAs in Canada where many are often unable to participate in community social activities because they are engaged in house chores, this could lead to social isolation and challenge the older adults’ sense of social connectedness and sense of belonging. Participating in community programmes enables RIOAs to engage with other older adults in group activities, such as day programmes, and meet and make new friends, as well as combat loneliness [39, 40]. Language is not just an issue of connecting with others to socialize and access services in general society; RIOAs also derive a sense of comfort from speaking their language because it reinforces a shared identity and a sense of belonging within their ethnocultural community and Canadian society [60]. Language proficiency opens doors for good and stable jobs, as well as opportunities to contribute to a retirement pension [15]. However, many RIOAs face linguistic challenges with Canada’s official languages of English and French, predisposing them to poverty and limiting their social connections to the general population [12]. Better availability of support for RIOAs could lessen vulnerability and enhance their social involvement in society to promote their wellbeing.

At the interpersonal level, the review highlights the older adults’ social contacts, support systems and the role of familial and community support, particularly ethnocultural groups, faith communities and settlement organizations, in facilitating RIOAs’ ability to form social relationships in Canada. Newly migrated older adults depend on the adult children who sponsored them and support their adult children with childcare. Many of the older adults’ children are very occupied with work and thus have limited time to provide support, such as transportation when needed, or relieve the older adults of duties such as childcare to enable them to attend social programmes. This dependence on their adult children could lead to abuse and the social isolation of RIOAs.

At the organizational level, we explored the interaction between the older adults and institutions. Many RIOAs experienced untoward challenges with the labour market, including deskilling and taking unstable jobs. Some continue to work despite reaching the Canadian retirement age of 65 years. Deskilling and working in low‐wage jobs adds to the high prevalence of poverty among RIOAs, indirectly impacting their social connectedness, sense of belonging and mental health [61, 79]. Mental health concerns, though prevalent among racialized populations, are underreported [57, 74]. This review’s findings on mental health align with those from a past review that reported the prevalence of mental health disorders, such as depression, was higher among Chinese older adults than the general older adult population in Canada [5]. The organizational level also includes access to services such as healthcare. RIOAs cannot easily access such services due to language barriers, racism, poverty and immigration rules. The findings of Grenier et al.’s [12] study align with those from this review that indicate racism affects RIOAs’ access to essential services and engagement with the general population.

At the community level, a key finding is that RIOAs feel like strangers in their own neighbourhoods. The neighbourhood can be a difficult place for many RIOAs to connect to people and feel accepted, particularly Black older adults, women of the Islamic faith and those facing language barriers [12, 60, 65, 81]. Volunteering and participating in community social programmes such as day programmes, community health centres and religious communities can provide social support and networking opportunities for RIOAs [65], thereby enhancing their social connectedness, sense of belonging and wellbeing. However, some RIOAs are hesitant to interact with the Canadian general population due to prior experiences of discrimination and racism, thus excluding many RIOAs in the community and contributing to their social isolation [60, 65]. Another reason for RIOAs’ challenges connecting with the community and feeling a sense of belonging is the difficulty in making trusted new friends and learning new cultural values that are different from what they have experienced most of their lives, as reported from a study conducted on international retired migrants from the Netherlands living in other destination countries [26].

Social isolation is detrimental to the older adult population, as those who are socially isolated are at greater risk of developing emotional and social loneliness [26, 39, 40] and Alzheimer’s disease [78]. Racial differences in the social connectedness and sense of belonging of RIOAs in Canada were not explored in the literature. However, a few studies focused on the social connectedness and sense of belonging of Arab, Black, Chinese and Filipino older adults in Canada. A previous scoping review on racialized older adults’ access to healthcare reported similar findings, with considerable literature found on Chinese and South Asian older adults’ social engagement in Canada; this finding could be due to these groups being the largest immigrant populations in Canada in the 1990s and the interest of researchers in reporting how the older adults in these groups struggle with connecting to the community [5]. Minimal research focussing on Central American or Japanese older adults’ social connectedness and sense of belonging in Canada was found during this review; members of these groups may experience fewer challenges and greater success integrating and socially connecting with Canada’s general population, but such speculation requires further research.

The last level, public policy, looks at how policies in the country impact RIOAs’ social inclusion and acceptance in the country. While RIOAs are not a homogeneous group, slight differences were found within the group regarding the impact of the environment on their social connectedness and sense of belonging in Canada. RIOAs with long residency (40 years or more) who are educated tend to be better engaged with the general population, which could be due to their financial security from pension benefits and friendships they have formed [15, 80]. Taylor et al. [38] conducted a study with older adults in the United States of America with findings that align with those noted here regarding the influence of financial security on RIOAs’ social engagement and sense of belonging in the community. The inequity many RIOAs encounter in Canada affects their social connections and negatively impacts their sense of belonging, as well as their wellbeing if unaddressed. Support in the form of transportation coupons, reduced fees to attend social programmes, grocery store coupons and health insurance for low‐income RIOAs will enable many older adults to overcome the challenges related to accessing services and connecting with the community that result from immigration and social services policies.

### 4.1. Implications

The findings of this review bring awareness to the personal and environmental barriers that impact the social connectedness and sense of belonging of RIOAs in Canada. RIOAs’ social engagement is affected by a combination of personal and layers of environmental factors that require a collaborative effort by gerontological service providers (i.e., nurses, geriatricians, social workers and others) to address. Educational and social support programmes that promote and enhance RIOAs’ social connections in Canada are needed. Furthermore, community member education could focus on the increasing population of ageing immigrant older adults and the need to accept them and support their participation in community programmes. Subsidizing fee‐based programmes for RIOAs who find them unaffordable will also facilitate enhanced connection to the community and increase the number of social contacts. Services supporting RIOAs may also consider hiring people who look like RIOAs to support their usage and integration. Inclusive policies providing support for RIOAs who are ineligible for social benefits in the country could be considered to reduce the isolation, financial and health challenges this population experiences. Provincial governments and healthcare providers may consider policy reforms that are more inclusive and cover uninsured and undocumented older adults, allowing them to access essential services such as healthcare, transportation and social services.

This scoping review revealed a gap in the literature; specifically, current gerontological studies in Canada are predominantly conducted among Chinese and South Asian communities, with few studies on Black and Arab RIOAs. Research conducted with other racialized groups, such as those who identify as Hispanic or Japanese, will contribute insights regarding their ageing experiences in Canada. Another identified research gap includes the limited studies on the impact of the physical environment, e.g., weather, space and neighbourhood, on RIOAs’ social connection in Canada. Moreover, many studies focused on the intrapersonal level (individual characteristics), interpersonal level (family members and friends) and organizational level (social structures including work, healthcare) of the SEM, with limited research on the community level (neighbourhood social cohesion) and public policy level (embedded societal cultures including discrimination in policies).

### 4.2. Limitations

Some articles might have been missed or excluded from this review as a result of the negotiation process employed to address conflicts. Additionally, articles may have been excluded because they were published more than 12 years ago, involved participants younger than 55 years or did not provide information about the country in which the study was conducted or the group of racialized people who participated. However, the findings from this review are mostly consistent with the findings of the articles deemed relevant to this review.

Although Canada is a multicultural country with two official languages, the scope of the review was limited to studies published in English, which may have excluded relevant literature in other languages. Moreover, the grouping of RIOAs as defined by Statistics Canada hides essential differences among racialized groups and complicates the systematic literature search in review studies [5]; as such, differences among various racialized groups in terms of social connectedness and sense of belonging in Canada were not explored. However, this review identifies gaps in the literature on racialized older adult populations in Canada and highlights the need for more disaggregated research that can inform targeted interventions and policies to support RIOAs.

## 5. Conclusion

The literature on RIOAs in Canada employs the terms social connectedness and sense of belonging interchangeably. However, this review considers social connectedness as racialized immigrant older adults’ social contact with people, while belonging is their subjective experience of happiness, close ties to people, reciprocal support, integration in and sense of belonging to the community and acceptance from the general population. Most studies on RIOAs were conducted in urban Canadian settings and focused on the social environment, particularly support from family members and friends. The findings from this review highlight RIOAs’ limited connection to the community and challenges encountered with finding employment due to immigration requirements, structural racism and language barriers, which contribute to the group’s experience of poverty in Canada. A major contribution of this scoping review is to highlight the impact of interrelated multiple environmental factors, such as poverty, culture, neighbourhood and immigration policies, on RIOAs’ social connectedness and feeling of belonging in Canada. Specific impacts from poverty occur due to life course experience, including their late settlement in the country and policies that limit their participation in the labour market. The limited social connections experienced by some RIOAs could contribute to a reduced sense of belonging and inadvertently lead to loneliness among the population. A gap in the literature is the few studies that considered how the physical environment (i.e., weather, space including parks, sidewalks, neighbourhood safety and cohesion) impacts RIOAs’ social connections and ageing in Canada, which future researchers could explore. In addition, the review identified limited studies on the social connectedness and sense of belonging of RIOAs from Arab, Black, Hispanic and Japanese backgrounds in the Canadian gerontological literature, indicating those groups should be included in future research.

## Funding

This study was supported by Social Sciences and Humanities Research Council of Canada Partnership Development Grant.

## Disclosure

The authors value equity, diversity and inclusion (EDI) in their research work and consider EDI in the formation of the team and hiring of research staff.

## Conflicts of Interest

The authors declare no conflicts of interest.

## Supporting Information

Additional supporting information can be found online in the Supporting Information section.

## Supporting information


**Supporting Information 1** 1. PRISMA flow chart.

**Supporting Information 2** 2. Search strategy in Ovid Medline.

**Supporting Information 3** 3. Data extraction table on articles that net the inclusion criteria for the scoping review.

**Supporting Information 4** 4. PRISMA‐ScR.

## Data Availability

All data applicable to the manuscript are provided as a supplementary document in the submission.
