# Supplementary material for: A Scoping Review on the Impact of the Environment on Racialized Immigrant Older Adults’ Social Connectedness and Sense of Belonging
Source: J Aging Res. 2026 Feb 20;2026:1089194. doi: 10.1155/jare/1089194 (PMC12921639; doi:10.1155/jare/1089194)
Supplement: Supplementary file 1 — Supporting Information 1 1. PRISMA flow chart. [file JARE-2026-1089194-s004.doc]

**
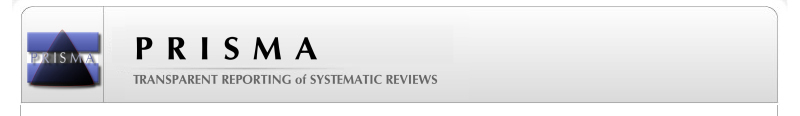
PRISMA 2009 Flow Diagram on Studies Identified Via Databases and Registers**

**Screening**

**Included**

**Eligibility**

**Identification**

Records identified through database searching

Ageline n=31, CINAHL n= 12, Embase n= 265, Ovid Medline n=397, APA Psychinfo n=195. Total n= 900

Additional records identified through other sources (such as Registers e.g. JBI & Cochrane) n = 0

Duplicate records removed (n=185)

Records after duplicates removed
(n = 715)

Records screened
(n =715)

Records excluded
(n =641)

Full-text articles assessed for eligibility
(n = 74)

Full-text articles excluded, with reasons (n=37): Age younger than 55; study on health and access to healthcare; racialized country not provided.
(n = 0 )

Studies included in review qualitative (n = 24)

Studies included in review

quantitative (n =12)

research synthesis (n=1)
