# Supplementary material for: A Scoping Review on the Impact of the Environment on Racialized Immigrant Older Adults’ Social Connectedness and Sense of Belonging
Source: J Aging Res. 2026 Feb 20;2026:1089194. doi: 10.1155/jare/1089194 (PMC12921639; doi:10.1155/jare/1089194)
Supplement: Supplementary file 2 — Supporting Information 2 2. Search strategy in Ovid Medline. [file JARE-2026-1089194-s002.docx]

Appendix 1: Scoping Review on the Impact of the Living Environment on Older Racialized Immigrants Social Connectedness Search Strategy and **and Sense of Belonging**

Ovid MEDLINE(R) ALL <1946 to February 15, 2023>

1 aging/ or exp Geriatrics/ or exp Aged/ or Health Services for the Aged/ or Senior Centers/ 3607610

2 (old age* or aging adult* or elder* or retiree* or ((old* or retired or senior) adj2 (adult* or people* or person* or individual* or wom#n or man or men)) or geriatr* or gerontolog* or senior citizen* or senescen* or sexagenarian* or septuagenarian* or octagenarian* or nonagenarian* or centenarian* or supercentenarian* or veteran*).tw,kf. 962509

3 1 or 2 4067443

4 racial groups/ or exp blacks/ or exp asians/ or exp ethnicity/ or exp "hispanic or latino"/ or "Ethnic and Racial Minorities"/ or Race Factors/ 271306

5 (racial* or race or ethnic* or minorit* or "people of colour" or "people of color" or Black or African or Asian* or Hispanic or Latino* or Indian* or Chinese or Japanese or Korean or Vietnamese or Afro-Carribean or Filipino or non-white or non-Caucasian or non-European or diaspora).tw,kf. 1251069

6 4 or 5 1320376

7 exp Residence Characteristics/ 77005

8 Environment Design/ or exp Social Environment/ 132626

9 interpersonal relations/ or intergenerational relations/ or friends/ or exp family/ 427957

10 (neighbour* or neighbor* or amenities or housing or community or communities).tw,kf. 871637

11 ((social ecolog* or living arrangement* or (home or social or physical or living or natural or built or cultural or economic)) adj2 (factor* or barrier* or obstacle* or environment*)).tw,kf. 146153

12 ((relationship* or connection*) adj2 (friend* or famil* or interpersonal or intergeneration* or inter-generation*)).tw,kf. 20147

13 7 or 8 or 10 or 11 1119927

14 Loneliness/ 5952

15 social adjustment/ or exp social discrimination/ or exp social inclusion/ or exp social isolation/ or social marginalization/ or social integration/ or social cohesion/ or interpersonal relations/ or intergenerational relations/ 133133

16 exp "Quality of life"/ 259545

17 Psychological well-being/ or Mental Health/ or exp Adaptation, Psychological/ 193528

18 (lonely or loneliness or alienat*).tw,kf. 15463

19 ((relationship* adj2 (friend* or famil* or interpersonal or intergeneration* or inter-generation*)) or "lack of contact" or social capital or (social* adj2 (connect* or cohesion or integration or disconnect* or acceptance or deprivation or isolat* or marginal* or limited or infrequent))).tw,kf. 53151

20 (life satisfaction or (quality adj2 life) or happiness).tw,kf. 387141

21 ((wellbeing or well-being or wellness or (mental* or emotional or social or psychological)) adj1 (health or hygiene or well* or unhealthy or disorder* or dysfunction*)).tw,kf. 409086

22 14 or 15 or 16 or 17 or 18 or 19 or 20 or 21 1055224

23 exp Canada/ 179490

24 (canadian* or canada* or canadien* or british columbia* or vancouver* or (Victoria* not Australia*) or alberta* or calgary* or edmonton* or saskatchewan* or regina* or saskatoon* or manitoba* or winnipeg* or ontario* or toronto* or ottawa* or McMaster or thunder bay* or London Health Science* or University of Western Ontario or Western University or (Queen* adj University) or quebec* or McGill or (new brunswick* not Chicago) or Moncton* or Fredericton* or prince edward island* or Charlottetown* or nova scotia* or Halifax* or Dalhousie or labrador* or newfoundland* or ((St or Saint) adj John*) or memorial university or nunavut* or Iqaluit* or northwest territor* or Yellowknife* or yukon* or Whitehorse*).ti,ab,hw,kf,jw. 492219

25 ((Hamilton not ((Hamilton adj New Zealand) or ((Chedoke or Hamilton or McMaster) adj4 (equation? or index* or inventor* or norwood or scale?)))) or (Hamilton adj Ontari*)).ti,ab,hw,kf,jw. 5582

26 (canadian* or canada* or canadien* or british columbia* or vancouver* or (Victoria* not Australia*) or alberta* or calgary* or edmonton* or saskatchewan* or regina* or saskatoon* or manitoba* or winnipeg* or ontario* or toronto* or ottawa* or hamilton* or thunder bay* or quebec* or montreal* or new brunswick* or Moncton* or Fredericton* or prince edward island* or Charlottetown* or nova scotia* or Halifax* or labrador* or newfoundland* or ((St or Saint) adj John*) or memorial university or nunavut* or Iqaluit* or northwest territor* or Yellowknife* or yukon* or Whitehorse*).ia,in. 1149221

27 23 or 24 or 25 or 26 1449830

28 3 and 6 and 13 and 22 and 27 504

29 limit 28 to yr="2013 -Current" 353

| **Mesh Words Used in Ovid MEDLINE** |
| --- |
| Aging/ or exp Geriatrics/ or exp Aged/ or Health Services for the Aged/ or Senior Centers/ |
| Racial groups/ or exp blacks/ or exp Asians/ or exp ethnicity/ or exp "Hispanic or Latino"/ or "Ethnic and Racial Minorities"/ or Race Factors |
| Residence Characteristics |
| Environment Design/ or exp Social Environment |
| Interpersonal relations/ or intergenerational relations/ or friends/ or exp family |
| Loneliness/ |
| Social adjustment/ or exp social discrimination/ or exp social inclusion/ or exp social isolation/ or social marginalization/ or social integration/ or social cohesion/ or interpersonal relations/ or intergenerational relations |
| "Quality of life" |
| Psychological well-being/ or Mental Health/ or exp Adaptation, Psychological |
| Canada/ |
|  |

**Exclusion and Inclusion Criteria**

**Inclusion criteria**

- Articles on racialized older adults
- Study was conducted in Canada
- Focused on social connectedness, belongings, loneliness, isolation, social capital
- Discuss aspects of the environment

**Exclusion criteria**

- Studies conducted outside Canada
- Studies on older adult caregivers, health conditions and their diagnoses
