# Supplementary material for: A Scoping Review on the Impact of the Environment on Racialized Immigrant Older Adults’ Social Connectedness and Sense of Belonging
Source: J Aging Res. 2026 Feb 20;2026:1089194. doi: 10.1155/jare/1089194 (PMC12921639; doi:10.1155/jare/1089194)
Supplement: Supplementary file 3 — Supporting Information 3 3. Data extraction table on articles that net the inclusion criteria for the scoping review. [file JARE-2026-1089194-s001.docx]

Table 3: Data Extracted and Categories across Social Ecological Model (SEM) Levels

| Study | Country | Setting/Context/Research Approach | Participant Characteristics | Description of Results |
| --- | --- | --- | --- | --- |
| Intrapersonal level   - Older adults’ values, preference, language , motivation, financial and immigration status, experience of grief, living context | | | | |
|  |  |  |  |  |
| Lai DWL, Surood S. 2013.  **Effect of service barriers on health status of aging South Asian immigrants in Calgary, Canada** | Canada | Calgary, Canada.  Quantitative | South Asian immigrants age 55+ living in Calgary N=220 | Cultural/religious incompatibility, language differences are common barriers to healthcare. Lack of transportation/lack of support from family also results in worse mental health. |
| Yoshino S. 2013.  **Ethnic variations in care of older adults in Canada** | Canada | Data comes from two surveys conducted by Statistics Canada.  Quantitative | Caregivers and responsibilities to the older adults | The results indicate Asian older adults, were more likely to receive care only from family and friend caregivers. |
| Tieu Y, Konnert CA. 2014.  **Mental health help-seeking attitudes, utilization, and intentions among older Chinese immigrants in Canada** | Canada | Local community organizations, churches and Chinese assisted living facilities in Toronto.  Quantitative. | Older Chinese adults N=149 and ages (55-95) | Chinese older adults are more likely to take care of their own problem rather than seek emotional support from a friend. 34% live in multigenerational household; 8% in assisted living. |
| Coloma RS, Pino FL. 2016.  **"There's Hardly Anything Left": Poverty and the Economic Insecurity of Elderly Filipinos in Toronto** | Canada | Elderly Filipinos in Toronto. Mixed-method | At least 65 years old, be Filipino ancestry and has a Canadian citizen or permanent resident, living in the GTA | 7 out of 10 Filipinos in the GTA live in a state of economic insecurity or poverty. Older adults isolated from social interaction because of caregiving role for grandchildren and financial dependence on sponsoring family. |
| Study | Country | Setting/Context/ Research Approach | Participant Characteristics | Description of Results |
| Intrapersonal level  Older adults’ values, preference, language , motivation, financial status | | | | |
| Luo H. 2016.  **Strengthening Social Capital Through Residential Environment Development for Older Chinese in a Canadian Context** | Canada | Seniors’ group in the Chinese community participated in a survey and focus group.  Mixed method | N=101 Chinese elders 65-85 in Canada, born outside of Canada | 2/3 older adults live with spouses, children, and grandchildren. 32% live alone. 76% wants to live in separate household from family.  Older adults encounter language barrier in mainstream living arrangement.  Ethnic housing less preferred due to reduce lighting, hygiene, and experience of financial abuse. |
| Brual JJ. 2017.  **Later-life Filipino immigrants in the greater Toronto area: A case study of health status and utilization of services** | Canada | Senior-centred community and health care services in Toronto. Mixed-methods (interviews and survey) | Older Filipinos (55+) living in the GTA | Language barriers and transportation issues are experienced. Population can experience physical isolation, ethnic segregation, and poor social connections. |
| Garcia Diaz L, Savundranayagam MY, Kloseck M, Fitzsimmons D. 2019.  **The Role of Cultural and Family Values on Social Connectedness and Loneliness among Ethnic Minority Elders** | Canada | Two cities in Ontario.  Quantitative | Sample 123 older adults ages 50-93 years. Average age 69years originally from Asia, Latin America, Black Canadians, Europeans, Middle Eastern. Born outside Canada. | 56% understand few words in English(language barrier). Lower income contributes to higher levels of loneliness in Canada. Seniors who value familism (solidarity of family members report higher level of loneliness as they do not want to receive support outside the family as it is considered disgrace to the family. |
| Study | Country | Setting/Context/ Research Approach | Participant Characteristics | Description of Results |
| Intrapersonal level  Older adults’ values, preference, language , motivation, financial and immigration status, experience of grief, living context | | | | |
| Tong CE, Sims-Gould J. 2021.  **Conducting a mixed-methods study with older adults in five languages: Lessons from the field** | Canada | South Vancouver/Sensors Advisory Council at South Vancouver. Questionnaires and interviews.  Mixed-methods | Participants (n=49) were foreign-born, over the age of 65, community dwelling | A multilingual way of using signs can help participants instead of assuming which language they speak. Certain phrases can be impossible to translate from one language to another. |
| McAlpine AA, George U, Kobayashi K, Fuller-Thomson E. 2022.  **Physical Health of Older Canadians: Do Intersections Between Immigrant and Refugee Status, Racialized Status, and Socioeconomic Position Matter?** | Canada | Statistics Canada 27^th^ Gss survey in 2013.  Quantitative | Age 55+ older immigrant adults of all ethnicities N=9011 | Refugees experience a higher chance of loneliness and discrimination in health care access. Racialized adults that have been discriminated against have a 50% higher chance of not reporting good health. |
| Fan Ng C, Northcott HC. 2015.  **Living arrangements and loneliness of South Asian immigrant seniors in Edmonton, Canada** | Canada | Local ethnic association and an immigrant settlement agency in Edmonton, Alberta. Mixed-method. | South Asian immigrants aged 60 years and above living in Edmonton, Canada. N=161 | Those who live alone are more likely to feel lonely than those who live with family. |
| Ferrer I. 2017.  **Aging Filipino domestic workers and the (in)adequacy of retirement provisions in Canada** | Canada | Filipino domestic workers in Canada. Qualitative research. | 6 women, ages 63-73 years old. | Some participants would still want to either work or receive a pension since they do not want to rely on their adult children. Intergenerational caring and reciprocation is a way of taking care of older adults. In return, they take care of the grandchildren, and some describe it as a “duty”. |
| Study | Country | Setting/Context/ Research Approach | Participant Characteristics | Description of Results |
| Intrapersonal level  Older adults’ values, preference, language , motivation, financial and immigration status, experience of grief, living context | | | | |
|  |  |  |  |  |
| Ferrer I, Brotman S, Grenier A. 2017b.  **The Experiences of Reciprocity among Filipino Older Adults in Canada: Intergenerational, Transnational, and Community Considerations** | Canada | Filipinos in Montreal, Canada. Qualitative research | 18 older people, 6 adult children, and 13 community stakeholders  Older adult participants ages 62-85 | Poverty has shaped caring relationships in pooling of resources and reciprocity; reliance on older adults to take care of grand children; older adults cut off from other social networks. |
| Ferrer I, Grenier A, Brotman S, Koehn S. 2017 a.  **Understanding the experiences of racialized older people through an intersectional life course perspective** | Canada | Racialized older people Filipino community in Montreal, Canada using qualitative research approach | 18 older adults, 6 adult children, and 9 community stakeholders living in Montreal Quebec | Participants still participate in intergenerational and transnational care across the border and with the family they have close tie with. Women primarily take on the caregiver role for grandchildren. |
| Gangopadhyay J. 2017.  **Aging Across Worlds: Examining Intergenerational Relationships Among Older Adults in two Cities in Transition** | India and Canada | Two cities, Saskatoon and Ahmedabad undergoing transformations in the form of urbanization, developmental policies and migration patterns. Mixed-methods | 20 South Asians over 60 years of residing in Saskatoon, Canada and 450in Ahmedabad, India. | Older immigrants in Saskatoon verbalized more independence, self-sufficiency, and financially independent. Elders in Ahmedabad have more social interactions and actively involved in many religious ceremonies. In Canada, the older adult’s kid's busy schedule correlates with their level of loneliness. |
| Study | Country | Setting/Context/ Research Approach | Participant Characteristics | Description of Results |
| Intrapersonal level  Older adults’ values, preference, language , motivation, financial and immigration status, experience of grief, living context | | | | |
| Koehn S, Baumbusch J, Reid RC, Li NKM. 2018.  **'It's Like Chicken Talking to Ducks' and Other Challenges to Families of Chinese Immigrant Older Adults in Long-Term Residential Care** | Canada | Two long term care (LTC) facilities in British Columbia (BC), Canada. Qualitative research. | BC LTC residents aged 65+ who spoke Cantonese as their first language n=295 | Racialized older adults in LTC experience inequity, racism, and ageism. Some Chinese carers said they felt that they were better off sharing their concerns with another (non-Chinese) member of the Family Council rather than attending themselves. |
| Zhang W. 2019.  **Perceptions of elder abuse and neglect by older Chinese immigrants in Canada** | Canada | The present study is based on a self-initiated project by an association of Chinese seniors in the Greater Toronto Area in Canada. Qualitative research. | Older Chinese immigrants (65+) in Canada n=12 | Emotional abuse has been experienced in the form of mistrust, and neglect (when the family isn’t around when they needed care). Lack of language skills and abuse are most common concerns discussed in the article. Canadian policy puts older racialized adults at more risk for abuse. |
| Salma J, Salami B. 2020a.  **"Growing Old is not for the Weak of Heart": Social isolation and loneliness in Muslim immigrant older adults in Canada** | Canada | Muslim communities in Edmonton. Qualitative approach | Older adults 55years and above; Sample size 67 older adults and 16 stakeholders. | Vulnerable group of older adults are late-in-life immigrants in the family re-unification category who do not receive benefits from Canadian government and had to depend on their sponsored family for 20 years. |
| Study | Country | Setting/Context Research Approach | Participant Characteristics | Description of Results |
| Intrapersonal level  Older adults’ values, preference, language , motivation, financial and immigration status, experience of grief, living context | | | | |
| Salma J, Salami B. 2020b.  "We Are Like Any Other People, but We Don't Cry Much Because Nobody Listens": The Need to Strengthen Aging Policies and Service Provision for Minorities in Canada | Canada | Stakeholders and older adults from local ethnocultural and religious organizations in Edmonton. Qualitative research. | 67 older adults and stakeholders from South Asian, Arab, and African Muslim communities in Edmonton, Alberta. | Muslim Immigrant women and lower-income older adults experience more loneliness. Finding older adults who speak the same language and share the same values is key to combating loneliness. |
| Foster N, Kapiriri L, Gringon M, McKenzie K. 2022.  **"but...I survived": A phenomenological study of the health and wellbeing of aging black women in the greater toronto area, Canada** | Canada | Greater Toronto Area(GTA). Participants were recruited through partnerships with community health centres (2), local churches (3), and various ethnic (20) and professional (2) associations. Qualitative research approach | Black women (n=27) age 55 and older living in the GTA social and physical health | Past trauma from racism, gender inequality, and abuse affects physical and mental health. Very few participants sought professional mental help assistance. |
| Zhang W. 2022.  **Perceptions and expectations of filial piety among older Chinese immigrants in Canada** | Canada | Chinese immigrants living in the GTA. Qualitative approach. | Chinese immigrants (65-84) in the GTA N= 46  Originally from Hong Kong, Taiwan, Mainland China | Some Chinese older adults reduced their expectations for children to care for them because of the burden. However, most value the emotional care and respect of their children. Their perception of filial piety remains the same while their expectation changes depending on finances, and family ties. |
| Interpersonal Level  Relationship between older adults and social contacts such as children, other family members, friends, and neighbors in Canada | | | | |
|  |  |  |  |  |
| Martin-Matthews A, Tong CE, Rosenthal CJ, McDonald L. 2013.  **Ethno-cultural diversity in the experience of widowhood in later life: Chinese widows in Canada** | Canada | Non-profit agency serving in the Greater Toronto Area/Chinese. Qualitative approach | **Chinese widows women age 69-93 in the GTA N=20** | Immediate family provides visits, daily interaction. Some older adults reported being lonely because children are busy working or outside the country in the US. |
| Park JM. 2014.  **The experience of elderly Koreans' Han and its implication for spiritual care: In the Canadian immigrant context** | Canada | Han as a concept signifies human suffering. Qualitative research | 10 elderly Koreans in Ontario age 65+. | This is a doctoral dissertation thus authors extracted data relevant to review question. Verbalizes that hardships are the most important Han experience (grief related to war, language barrier, poverty), family conflicts between husband and wife and church conflicts can happen which in turn affect participants’ mental health. |
| Da W-W, Garcia A. 2015.  **Later life migration: Sociocultural adaptation and changes in quality of life at settlement among recent older Chinese immigrants in Canada** | Canada | London Ontario. Qualitative research. | Older Chinese women age 55-75+ immigrants living in Canada n=31 | Important social support networks are churches, ESL classes, and other social programs. However, transportation can be a barrier to attend. A lot of older adults are their grandchildren's full-time caregivers, part of the reason is the dependency rule. |
| Study | Country | Setting/Context/ Research Approach | Participant Characteristics | Description of Results |
| Interpersonal Level  Relationship between older adults and social contacts such as children, other family members, friends, ethnocultural groups, and neighbors in Canada | | | | |
| Matsuoka, A. K. 2015.  **Ethnic/Racial Minority Older Adults and Recovery: Integrating Stories of Resilience and Hope in Social Work** | Canada | Metropolitan city in Canada  Participants were Japanese Canadian older adults in Canada. First generation Japanese speaking Canadians. Qualitative research. | Workshop for older Japanese-Canadians age 64-89 in a metropolitan city in Canada N=8 | The morbidity of mental illness is higher among racialized older adults compared to Canadian-born adults. Participants find workshops beneficial to their self-worth, optimism, and confidence  Older adults (OA)gained knowledge of what to do to keep healthy- go for a walk, sing with others, hear others, and reaffirmed own worth. |
| Koehn SD, Mahmood AN, Stott-Eveneshen S. 2016.  **Quality of Life for Diverse Older Adults in Assisted Living: The Centrality of Control** | Canada | Assisted living (AL) in Canada.  Qualitative research approach | Diverse older adults Site 1 (50 units) is targeted to South Asians, Site 2 (60 units) is targeted to Chinese, and Site 3 (58 units) caters primarily to a mainstream Anglo Canadian clientele. | The older adults benefited from speaking their language and eating their ethnic food.  Ethnocultural assisted living reported more people are compatible as they ate and spoke the same language. |
| Charpentier M, Queniart A. 2017.  **Aging experiences of older immigrant women in Quebec (Canada): From deskilling to liberation** | Canada | Immigrant women from Quebec who took part were split into 18 focus groups covering 4 continents and 17 different countries. Qualitative research | 83 older women immigrants between 58 and 88 years old  From Arab, Africa, Haitian, Japanese, Chinese, Portuguese, Romanians | The difficulty in integrating into the Labour market and discrimination for years are present among these women. Many women experience deskilling Many of these women are poor and single, and must overcome everyday challenge (aging alone and loneliness). Many reported a sense of “freedom” and liberation from the social standards. |
| Study | Country | Setting/Context/ Research Approach | Participant Characteristics | Description of Results |
| Interpersonal Level  Relationship between older adults and social contacts such as children, other family members, friends, ethnocultural groups, and neighbors in Canada | | | | |
| Ferrer I, Lee Y, Khan MN. 2020.  **Understanding the Lived Experiences and Financial Realities of Older Immigrants** | Canada | Immigrants communities "racial"/ethnocultural affiliation in the city of Calgary using photovoice design.  Qualitative research approach | 11 older immigrants (60 and over) | Older immigrants are vulnerable to financial abuse from loan agencies, and family members because of language barriers  Older adults shared OAS, CPP are not enough for home repairs such as leaking faucets and stoves, washing machine, faulty ceilings.  The older adults help at home and teach their grandchildren cultural heritage or knowledge while depending on adult children. |
| Hepburn, S. (2020). Retirement time & the temporalities of the migratory life course. | Canada | Greater Toronto Area  Qualitative research. | 20 Jamaican Canadian older adults ages 60 years and above | Diversity of older adults with many who held precarious jobs and unable to contribute to Canada pension plan from early earnings. Those with stable jobs contributed to pension plan and were safe for retirement. Some older adults worked after retirement to offset senior poverty. Unable to afford -in support to assist older adult to attend day program as they need help to go for the program. Older adults at risk of isolation. |
| Lai DWL, Li J, Ou X, Li CYP. 2020.  **Effectiveness of a peer-based intervention on loneliness and social isolation of older Chinese immigrants in Canada: a randomized controlled trial** | Canada | Chinese communities, independent living and assisted living facilities for older Chinese in Calgary.  Quantitative research. | 60 Chinese immigrants aged 65 years and older in Calgary. Design: RCT | loneliness using the De Jing Loneliness scale and social support network using the Lubben Social Network Scale, life satisfaction and resilience were also measured. The group that received intervention (peer-based intervention ) experienced higher levels of happiness. |
| Study | Country | Setting/Context/ Research Approach | Participant Characteristics | Description of Results |
| Interpersonal Level  Relationship between older adults and social contacts such as children, other family members, friends, ethnocultural groups, and neighbors in Canada | | | | |
| Dhillon S, Humble AM. 2021.  **The sociocultural relationships of older immigrant Punjabi women living in Nova Scotia: Implications for well-being** | Canada | Recruitment in large temples in Nova Scotia using convenience sampling. Qualitative research | Punjabi women age 65-68 years old living in Nova Scotia n=5 | Difficulty merging into Canadian community due to language barrier was experienced which leads to a feeling of loneliness and lack of freedom. Dependent on family members for doctors’ appointments and driving takes away freedom.  Participants reminiscence about home country (India). No community here in Canada as no one knows them; everyone is a stranger to them. |
| Herman L, Walker R, Rosenberg M. 2021.  **Age-Friendly Communities and Cultural Pluralism: Examining Saskatoon's Chinese-Canadian Social Enclave** | Canada | Chinese Canadian older adults in Saskatoon. Qualitative research. | Older adults who are Chinese Canadian age 55 years and older n=20 | Many Chinese Canadian rely on social enclave as support so they choose to reside in places that give them more proximity linguistically and culturally. |
| Puplampu, V, Pierson, J, Oba, F, Weeks, L, Gawdun, J, White, J 2024.  Older Black adults’ social capital during COVID-19 and its impact on mental well-being. | Canada | Saskatoon, Saskatchewan. Qualitative research | 22 older Black adults ages 65 and four community organization workers | The older adults appreciated Canada and indicated it is a blessed country. Participants identified limited social programs that met the needs of older Black adults. inter |
| Study | Country | Setting/Context/ Research Approach | Participant Characteristics | Description of Results |
| Organizational Level: Older adults’ interaction with institutions such as work, health care settings. | | | | |
| Ferrer I, Brotman S, Koehn S. 2022.  **Unravelling the Interconnections of Immigration, Precarious Labour and Racism Across the Life Course** | Canada | The intersectional perspective holds the potential to situate personal stories within wider social processes. Qualitative research | Sample was 19 older immigrants in Canada living in Montreal, Quebec and Vancouver, British Columbia. | Women primarily take on the caregiver role for grandchildren.  Accreditation process as systemic racism. Work place abuse such as live in caregiver or domestic worker. |
| Koehn S, Ferrer L, & Brotman, S. 2022. Between loneliness and belonging: narratives of social isolation among immigrant older adults in Canada. | Canada | Structured life-story narratives and photovoice from four case studies of immigrants older adults in Vancouver.  Qualitative research approach. | 4 Korean, Pakistan, and Filipina older adults ages 62 and 77 living in Montreal and Vancouver. | Language barriers and failure of employers to recognize educational credentials forced older immigrants into service industry, which contributes to their low income.  Older immigrants’ ability to access social programs depend on their children and grandchildren’s schedule. Limitation in mobility due to snow and transportation. |
| Lin S. 2022.  **Access to health care among racialized immigrants to Canada in later life: A theoretical and empirical synthesis** | The study aim was to synthesize literature to explain what and how social-cultural determinants lead to barriers in accessing healthcare among racialized immigrants. | Health care access in multiple cities across Canada in the racialized immigrants population.  Systematic review. | Databases such as PubMed, and Web of Science were searched for peer-reviewed studies in English using search strings such as racial, minorities, health care, Canad*. included. | 35 articles were used. Linguistics and cultural practices in healthcare are barriers for immigrants. Racism and stigmatization and oppression of immigrants, health policies and immigration policies also play a role in access to healthcare. |
| Su C, Yang L, Dong L, Zhang W. 2022.  **The Psychological Well-Being of Older Chinese Immigrants in Canada amidst COVID-19: The Role of Loneliness, Social Support, and Acculturation** | Canada | Chinese older immigrants in Canada.  Quantitative research | 160 older Chinese immigrants ages 65-75 | Individuals who experienced social isolation and loneliness are less likely to attend social activities, more likely to show depressive symptoms and likely unsatisfied with their life. |
| **Community Level: RIOAs Experience in the Neighbourhood**  Includes these studies listed above: Brual; Dhillon and Humble; Charpentier & Queniart; Gangopadhyay ; Herman et al. ; Koehn et al.; Lai & Surood ; Martin-Matthews et al. ; Ojembe et al.; Salma & Salami | | | | |
| Ojembe, B., Kapiriri, L., Griffin, M., & Schomans, A. F. (2024). “You’re not understood, and you’re isolated”: A narrative account of lowliness by Black older adults in Ontario, Canada. | Canada | Ontario. Narrative inquiry. Qualitative research. | Thirteen Black older adults (BOAs) 55years and older | BOAs experience loneliness due to relocation, loss of significant relationships, disability, poor health, and lack of support. Experience of racism contributed to depression. Neighbourhood played key role in loneliness or belonginess. Finding religious institutions as a way to connect to other community members. |
| Policy Level: influence of the societal rules, policies, culture, immigration laws on older adults | | | | |
| Coloma RS, Pino FL. 2016.  **"There's Hardly Anything Left": Poverty and the Economic Insecurity of Elderly Filipinos in Toronto** | Canada | Elderly Filipinos in Toronto. Mixed-method | At least 65 years old, be Filipino ancestry and has a Canadian citizen or permanent resident, living in the GTA | 7 out of 10 Filipinos in the GTA live in a state of economic insecurity or poverty. Older adults isolated from social interaction because of caregiving role for grandchildren and financial dependence on sponsoring family |
| Zhang W. 2019.  **Perceptions of elder abuse and neglect by older Chinese immigrants in Canada** | Canada | The present study is based on a self-initiated project by an association of Chinese seniors in the Greater Toronto Area in Canada. Qualitative research. | Older Chinese immigrants (65+) in Canada n=12 | Emotional abuse has been experienced in the form of mistrust, and neglect (when the family isn’t around when they needed care). Lack of language skills and abuse are most common concerns discussed in the article. Canadian policy puts older racialized adults at more risk for abuse. |
| Ferrer I, Lee Y, Khan MN. 2020.  **Understanding the Lived Experiences and Financial Realities of Older Immigrants** | Canada | Immigrants communities "racial"/ethnocultural affiliation in the city of Calgary using photovoice design.  Qualitative research. | 11 older immigrants (60 and over) | Older immigrants are vulnerable to financial abuse from loan agencies, and family members because of language barriers  Older adults shared OAS, CPP are not enough for home repairs such as leaking faucets and stoves, washing machine, faulty ceilings.  The older adults help at home and teach their grandchildren cultural heritage or knowledge while depending on adult children. |
| Salma J, Salami B. 2020a.  **"Growing Old is not for the Weak of Heart": Social isolation and loneliness in Muslim immigrant older adults in Canada.** | Canada | Muslim communities in Edmonton. Qualitative approach | Older adults 55years and above; Sample size 67 older adults and 16 stakeholders. | Vulnerable group of older adults are late-in-life immigrants in the family re-unification category who do not receive benefits from Canadian government and had to depend on their sponsored family for 20 years. |

Please note studies may fall under more than one level on the socioecological model. To avoid repetition on the table, we entered studies in one level based on where they were most used in the manuscript.
